# Being in the Past and Perform the Future in a Virtual World: VR Applications to Assess and Enhance Episodic and Prospective Memory in Normal and Pathological Aging

**DOI:** 10.3389/fnhum.2020.00297

**Published:** 2020-08-04

**Authors:** Azzurra Rizzo, Giuditta Gambino, Pierangelo Sardo, Valerio Rizzo

**Affiliations:** Department of Biomedicine, Neuroscience and Advanced Diagnostic, Università Degli Studi di Palermo, Palermo, Italy

**Keywords:** aging, pathological aging, virtual reality, episodic memory (EM), prospective memory (PM), assessment, cognitive training, cognitive impairment

## Abstract

The process of aging commonly features a gradual deterioration in cognitive performance and, in particular, the decline of memory. Despite the increased longevity of the world’s population, the prevalence of neurodegenerative conditions, such as dementia, continues to be a major burden on public health, and consequently, the latest research has been focused on memory and aging. Currently, the failure of episodic and Prospective memory (PM) is one of the main complaints in the elderly, considered among the early symptoms of dementia. It is therefore increasingly important to define more clearly the boundaries between normal and pathological aging. Recently, researchers have begun to build and apply Virtual Environments (VE) to the explicit purpose of better understanding the performance of episodic and PM in complex and realistic contexts, with the perspective of further developing effective training procedures that depend on reliable cognitive assessment methods. Virtual technology offers higher levels of realism than “pen and paper” testing and at the same time more experimental control than naturalistic settings. In this mini-review article, we examine the outcomes of recently available studies on virtual reality technology applications developed for the assessment and improvement of episodic and/or PM. To consider the latest technology, we selected 29 articles that have been published in the last 10 years. These documents show that VR-based technologies can provide a valid basis for screening and treatment and, through increased sensory stimulation and enriched environments reproducing the scenarios of everyday life, could represent effective stimulating experiences even in pathological aging.

## Introduction

Memory has been for centuries an intriguing field of brain research since it is a biologically essential function to the survival of almost all species (Bisaz et al., 2014). Memory is defined as the ability to acquire, process, store, and retrieve information (Fietta and Fietta, 2011). The remembering process is not a monolithic entity, but memory can be categorized and sub-categorized following many domains (Squire and Zola, 1996; Purves et al., 2001; Plescia et al., 2014). Conceivably due to the increasing human life expectancy and the growing incidence of severe diseases that can induce neuronal degeneration and alter neuronal excitability (Carletti et al., 2016, 2017; Jaul and Barron, 2017; Park and Festini, 2017), memory has been considered a core feature to study upon normal and pathological aging processes. Most people report some early age-related memory impairments since the age of 60, especially in longitudinal studies (Nilsson, 2003; Rönnlund et al., 2005). Even earlier, by the age of 30, the decline of some cognitive functions was evidenced in cross-cutting studies (Park et al., 2002). Nevertheless, specific memory abilities that do not entail the conscious recollection of previously experienced material seem not to be altered with normal and pathological aging (Mitchell and Bruss, 2003; Ballesteros and Reales, 2004). Classically, aging has been linked to neuronal loss independently of the brain region involved (Coleman and Flood, 1987). However, research involving healthy adults indicates that normal aging is always associated with morphological alterations in neurons belonging to structures involved in cognition (Tisserand and Jolles, 2003). Not all cognitive abilities are affected by aging, but impaired memory skills are generally reported by the elderly and give rise to bitter complaints (Craik, 2008).

Memory has been classified by time direction (Maylor, 1993): retrospective memory refers to the ability to retrieve past information. Focusing on episodic memory (EM) refers to long-term memories including specific information such as time, location, or perceptual details as well as the connection of multidimensional information (Tulving, 2002). Currently, the alteration of EM is considered the main early symptom of dementia (Gold and Budson, 2008), though, it is also common in healthy aging, therefore its failure is not specific of pathological aging (Nilsson, 2003; Rönnlund et al., 2005; Craik, 2008). Prospective memory (PM), on the other hand, is the ability to remember to execute previously planned actions and can be defined as “remembering to remember,” thus referring to the future, for example when you have to remember to take a drug at a certain time. Some studies suggest that the onset of pathological aging determines more difficulty in PM (Huppert et al., 2000). However, potential errors in PM may be associated with considerable risks (Smith et al., 2000; Maylor et al., 2002), for example forgetting to turn off the gas.

Considering this context, it is essential to differentiate normal and pathological aging, that is when aging brings about complications due to the presence of diseases such as Alzheimer’s disease (AD), Mild Cognitive Impairment (MCI), Parkinson’s Disease (PD), as well as other dementias (Hedden and Gabrieli, 2004; Craik, 2008) or diabetes type 2 (Redondo et al., 2016). Indeed, the progressive impairment in executive functions and memory processes in healthy adults could be exacerbated by the concomitant presence of common chronic diseases. Questions arise about if it is possible and how to inhibit pathological and non-pathological memory loss, especially considering the recent discovery of the ability of the nervous system to reconstruct cellular synapses upon interaction with enriched environments (Barak et al., 2013). A huge number of studies on the field have led to a better molecular understanding of different types of memory. Although, the most reliable way to assess memory processes in normal and pathological aging is still intensely debated.

Classic memory tests using paper and pencil or computer systems for the evaluation of EM usually require older adults to remember static stimuli, therefore, they may not provide sufficient detail for predicting patients’ daily difficulties in different dynamic environments. Some studies have argued that neuropsychological assessments should provide a good degree of similarity to daily life tasks since the lack of ecological validity can negatively affect predictions about patient’s memory failures (Schultheis et al., 2002; Parsons and Rizzo, 2008). Episodic retrieval, for example, requires information about central and perceptual details, space-time contextual elements, and the binding of this multidimensional information (Abichou et al., 2019). Similarly, in everyday life, people present motivational aspects and adopt strategies for coding daily intentions that are difficult to probe through classic tests. Moreover, these tests measure memory components in isolation and failing to offer a comprehensive understanding of their operation (Tulving, 2002). Even naturalistic observation is not always an effective solution due to a series of difficulties including problems of standardization, control of the stimuli and distractors, economic costs to physically build the observation environments, as well as security problems.

In this regard, an outstanding advantage could be posed by the usage of virtual reality (VR) that evaluates memory consolidation by interacting with an enriched everyday environment, which guarantees both laboratory analytical control and precise assessments of how memory and other cognitive processes operate. The main goal of VR is to allow the patient to undertake specific tasks through artificial sensory stimulation and the illusion of being in an interactive environment perceived as a real place (Mantovani and Riva, 1999; Riva et al., 2007; LaValle, 2019). This experimental application could be reconducted to the heterogeneous family of Embodied Cognition theoretical approaches claiming that the physical properties of the human body, especially perceptual and motor systems, must be considered essential factors for the development and functioning of a cognitive system and could modulate learning and memory formation (Madan and Singhal, 2012).

The concepts of immersion and presence, related to VR, can better describe the experience from the user’s physical and psychological point of view. The immersion refers to the physical configuration of the interface of a VR application: the number and range of sensory and motors channels connected to the system determine the “immersiveness,” ranging from Non-Immersive (NI) systems on desktop computers to fully immersive systems. This distinction is based on how much the user can perceive the outside world during the virtual simulation (LaValle, 2019). The fully immersive types are indeed characterized by the use of a head-mounted display (HMD) in which a high-fidelity graphic screen is mounted in front of one’s eyes with separate lenses for each eye. The interaction in this type of virtual reality is controlled by tracking the movement of the head in combination with a computer system, therefore when users move their head to look around, they consequently move their visual field within the virtual environment 360 degrees. The presence, on the other hand, is defined as “being in there” and occurs when the subject experiences an illusion of non-mediation in his space of action and acts as he would if the medium were not present (Mantovani and Riva, 1999; Riva et al., 2007). Presence is a subjective “response” to a system that has a certain level of immersion (Sanchez-Vives and Slater, 2005) people react and act on it as if they were real. This response is on many levels, ranging from unconscious physiological processes (cerebral, cardiac, skin, etc.) through to deliberate volitional behavior (Slater et al., 2009). In particular, the ability to induce the sense of presence seems to have positive effects on attention and involvement and consequently is very relevant in the evaluation of memory (Sutcliffe et al., 2005; Makowski et al., 2017).

In this review article, we aimed to explore the most recent evidence about the ability to evaluate EM and PM, as well as to stimulate improvements in both normal and pathological aging, through representative and significant examples of applications in VR.

## Methodology

Initially, a systematic online bibliography search was carried out through the following profile databases: Web of Knowledge, ScienceDirect, PubMed, and Google Scholar, on the date of June 2019. We used the following core search terms and their combinations: VR or virtual environment, prospective memory or PM, EM or EM; and the following as additional search terms with “Xor” combinations: assessment, cognitive training, aging, pathological aging, cognitive impairment. Also, to get a broader and more complete view of the topic we have included studies on young adult subjects. Second, a selection of relevant articles was limited to the period 2009–2019 to obtain information mainly about outcomes referring to the latest technology.

**Figure 1:**
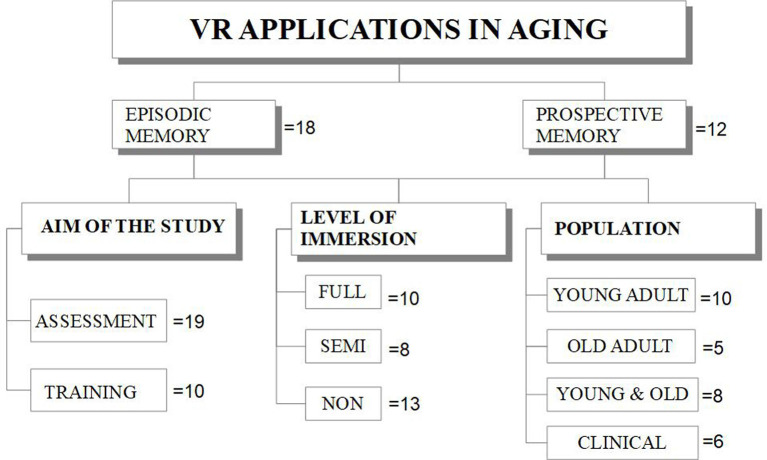
A schematic representation of the studies selected in this review article, based on the inclusion criteria, on Virtual Reality (VR) applications in Episodic Memory (EM) and Prospective Memory (PM). The articles reviewed were subdivided based on the aim, the level of immersiveness, and the population observed. A total of 17 studies in EM and 12 in PM (one of which was included in both the groups because provided data on EM and PM).

The references selected were included in the review in case the following criteria were met as shown in Figure 1: research on the impairment of EM or PM in the aging; description of VR methodologies for assessment or training; a clear description of VR tools that determine the related level of immersion. Overall, 29 studies have been identified and summarized in this mini-review article and classified as in Table 1.

**Table 1:** Virtual reality (VR) studies in aging classified according to contribution in Episodic Memory (EM) or Prospective Memory (PM), kind of intended purpose divided into Assessment (AS) or Training (TR), level of immersion divided into Full-Immersive (FI) or Semi-Immersive (SI) or Non-Immersive (NI); Experimental subjects (ES); the presence of Information about Cybersickness (IC); type of Environment; the number of Training Session (TS); navigation Time and Main results.

	Articles included	FI	SI	NI	AS	TR	ES	IC	Environment	TS	Time	Main results
**EM**	Sauzéon et al. (2012)						Young adults	**X**	Apartament		N/A	Active exploration increases hippocampal activity and improves spatial learning
	Plancher et al. (2012)						Older adults and AD patients					Interaction and planning affect spatial memory/worst memory performance for factual information
	Plancher et al. (2013)		**X**		**X**		Young adults		Urban		N/A	Interaction and planning affect spatial memory/worst memory performance for factual information
	Taillade et al. (2013)						Young and healthy older adults	**X**	Urban		10–15′	Worse spatial learning in motor control associated with executive functions
	Jebara et al. (2014)						Young and healthy older adults		Urban		N/A	Better recall of prospective intentions rather than retrospective in mild AD
	Plancher et al. (2018)						Young adults		Urban		20–25′	Concurrent activity (WM) negative impacts on long-term memory of central information
	Clemenson and Stark (2015)						Young adults		Angry bird Game; Super Mario 3d world	28	N/A	3D spatial aspect improves hippocampal dependent behavior
	Makowski et al. (2017)						Young adults		Avangers movie	1	N/A	Emotions and higher levels of presence associated with better memory
	Serino et al. (2017)		**X**		**X**		Older adults and AD patients		Urban	10	20′	Spatial performance improvement in AD/spatial decision making improvement in healthy subjects
	West et al. (2017)						Healthy older adults		Super Mario 64; Super Mario Galaxy	120	30′	3d spatial aspect improves hippocampaldependent behavior
	Bakdash et al. (2008)						Young adults		Urban		20′	Decision-making positively affect EM encoded vs. control
	Bergouignan et al. (2014)						Young adults		Room		N/A	Depersonalization states compromise the coding of EM
	Parsons and Barnett (2017)		**X**		**X**		Young and healthy older adults	**X**	Grocery store		15–20′	Virtual Shop construct validity
	Corriveau-Lecavalier et al. (2020)						Young and healthy older adults	**X**	Grocery store		N/A	Construct validity of the virtual shop/higher levels of motivation than traditional memory test
	Optale et al. (2010)	**X**				**X**	Healthy older adults		Familiar setting (family home, park, ecc)	36 + 24	15′	Improvement in general cognitive functioning and in long-term memory
	Serino et al. (2015)			**X**	**X**		Healthy older adults and AD/aMCI patients		Room		N/A	Deficiencies of aMCI and AD patients in storing hetero-centric independent representational memories/deficiencies of AD patients in synchrony
	Abichou et al. (2019)						Young and healthy older adults	**X**	Urban		N/A	EM consolidation after a period of sleep
	Plechatá et al. (2019)	**X**		**X**	**X**		Young and healthy older adults	**X**	Supermarket		10′	Elderly lower performance in HMD
**PM**	Nolin et al. (2013)						MCI patients and older adults		Apartment		N/A	Positive correlation between PM Virtual task and MoCA test
	Dong et al. (2017)						Young adults		Shopping streets		35′	Better correlation of virtual test with PM daily memory compared to desktop test
	Parsons and Barnett (2017)		**X**		**X**		Young and healthy older adults	**X**	Grocery store		15–20′	Virtual Shop construct validity
	Ouellet et al. (2018)						Young and healthy older adults	**X**	Grocery store		15′	Ecological and construct validity/Greater difficulty in virtual pointer technique in older adults compared to young adult
	Trawley et al. (2014)						Young adults		Office building	1	12′	Cue saliency increases attentional load on PM
	Yip and Man (2013)						ABI patients	**X**	Urban	12	35- 40′	Improvements in immediate recall of PM tasks performed by participants
	Debarnot et al. (2015)			**X**		**X**	Healthy older adults		Urban	1	30′	Improvement of the excitatory stimulation (Itbs) of Frontopolar cortex in event-based PM
	Mioni et al. (2015)						PD patients and older adults		Virtual day	2	15–20′	Emotionally-related improvement in PM performance
	Rose et al. (2015)						Healthy older adults		Virtual day	20	40–60′	Better scores in performing realistic PM tasks and IADL
	Gonneaud et al. (2014)		**X**		**X**		Healthy older adults		Urban		N/A	Link-EB task induce better performance than no link-EB and TB tasks
	Lecouvey et al. (2019)						Older adults and mild AD patients					Better recall of prospective vs. retrospective components of intentions in AD patients
	Dong et al. (2016)	**X**		**X**	**X**		Young adults		Shopping streets		N/A	Immersive VR induces prefrontal cortex activity in the BA10

## VR for Episodic Memory in Aging

Within Virtual Environments (VE), participants can be immersed in scenarios that represent different everyday situations such as virtual apartments (Sauzéon et al., 2012), grocery stores (Parsons and Barnett, 2017; Plechatá et al., 2019; Corriveau-Lecavalier et al., 2020) or city (Plancher et al., 2012, 2013, 2018; Abichou et al., 2019). This gives the chance to implement simple tasks to assess the versatile nature of EM in ecological situations in a rich and specific space-time context.

Plancher et al. (2018) analyzed the role of working memory (WM) while building an episodic trace, through an on-screen projected urban virtual environment. They reported that the memory of central information was altered by simultaneous tasks and that the memory of the temporal context and binding was compromised only upon the performance of a competing visuospatial activity. To the purpose of testing WM’s key role in consolidating the EM, participants were asked to explore the environment using a steering wheel, a gas pedal, and a brake pedal. At the same time, a secondary numerical task interfering with the phonological cycle (e.g., storing the number of garbage containers in the path) and a secondary visuospatial task (e.g., memorizing the spatial model of containers) were applied to predict that secondary activities performed during learning would interfere with coding and resulted in altered memory performances.

One of the main advantages of VEs is, indeed, the ability to precisely model and control the environment itself according to the requirements decided by the experimenter, avoiding possible problems of building real scenarios (Sauzéon et al., 2012). Due to the extreme adaptability of this technique, EM in VE has already been tested in clinical contexts (Plancher et al., 2012; García-Betances et al., 2015; Serino et al., 2015, 2017). To compare VR memory tasks with traditional neuropsychological tools for its evaluation, Plancher et al. (2012) conducted a study on healthy participants, patients with amnestic MCI (aMCI) and with mild Alzheimer’s. The experimental groups were asked to store as much information as possible during active and passive browsing conditions. The virtual task allowed characterizing the different cognitive profiles of the three populations and the authors found that spatial allocentric memory assessments discriminated against patients with aMCI from controls. Nevertheless, after active exploration of the VE, all participants, including patients with aMCI and AD, retrieved significantly better both central and allocentric spatial information and the process of binding. As pointed out by the authors, these results about active exploration are particularly promising because they provide support for the feasibility of VR as an effective non-pharmacological tool to promote neuroplasticity and neural reorganization in patients with AD.

Preclinical studies on aging have shown that immersion in enriched environments may drive long-term enhancement of the activity of the hippocampus and changes in memory-associated brain regions inducing structural changes in animal models (Harvey et al., 2009). Clemenson and Stark (2015) discovered that young adults trained with Super Mario 3D showed better spatial and EM performance dependent on hippocampus activity, compared to people trained in a 2D-controlled game. More recently, West et al. (2017) proposed the same 3D platform training applied to an elderly population reporting increases in gray matter thickness in brain regions known to be implicated in cognitive-related decline. Also, it was suggested that a greater feeling of presence improves the effectiveness of VR applications (Optale et al., 2010). It was found that higher levels of presence were associated with better factual memory and the impact of the emotional stimulus was mediated by a sense of presence (Makowski et al., 2017).

As pointed out by Repetto et al. (2016), besides environmental enrichment in VR research, two main aspects may be advantageous in the context of EM’s study. The first is that VR allows exploration from an egocentric point of view (Bergouignan et al., 2014; Serino et al., 2015, 2017); i.e., Bergouignan et al. (2014), using an out-of-body-induced illusion, have reported that an accurate EM encoding is favored by the perception of the world from the perspective of one’s own body. Second, VR enables active exploration of the environment. However, comparisons of active and passive navigation showed contradictory results with both negative (Taillade et al., 2013) and positive effects of active navigation (Sauzéon et al., 2012; Plancher et al., 2013).

Employing a similar paradigm to Plancher et al. (2013), Jebara et al. (2014) assessed the performance of a sample population of young adults and seniors in a virtual city projected on a screen. Four interaction conditions were included: “passive” (passengers in a virtual car cannot choose directions and route), “itinerary control” (passengers can choose), “low control” (driver move the car on rails) and “high control” (driver choose also direction). Better scores in EM (*what—where—when and binding*) in both young and old groups were obtained only in the conditions of choosing the route and low navigation control. This suggests that EM performance benefits from multimodal coding, through the enrichment of motor interaction and that it has been improved by active navigation when it is not too expensive in terms of attention efforts. According to some authors (Bakdash et al., 2008; Sauzéon et al., 2012; Jebara et al., 2014), active navigation may require additional cognitive resources that are not fully available for the coding process. Consequently, inconsistent results on memory performance related to the active-passive navigation may be due to differences in the manipulation of sensorimotor stimulation and its confusing effects on cognitive activity, as shown by several studies reporting worse memory performance caused by split attention (e.g., Craik et al., 1996). Plancher et al., 2013, have shown that driving in a VR while encoding information can be considered as a double task in which motor control can impact factual memory.

However, although these results suggest that active navigation VR training may have a beneficial effect on EM, it should be noted that older adults perform worse than young people, particularly in binding scores. This age-related effect noted in the low control condition encourages greater attention from research on the elderly regarding the complexity of the motor task which would risk having diametrically opposite effects on memory.

Lastly, as the use of HMD spreads, the effects of active and passive navigation must also be investigated in fully immersive environments especially for elder persons who are not familiar with technology and not used to handle it.

## VR for Prospective Memory in Aging

A growing number of studies on memory have focused on its prospective side, but uncertainties remain regarding the characteristics of PM impairments. It is still to be fully unveiled the influence of the executive functions, the life-span development of prospective remembering and the age effects, the underlying mechanism involved in event-based or in time-based PM task and the role of motivational aspect (Kliegel and Martin, 2003). Compared to traditional laboratory paradigms, virtual reality creates realistic tasks for the evaluation of the PM, increasing the variety of possible actions to perform. This allows measurements of multiple cognitive processes involved and to systematically control interactive stimuli with immediate feedback on performance through sensory modalities. Nolin et al. (2013) exploited an eMagin Z800 immersion target on a population of older adults with MCI vs. healthy controls in an urban environment. This VR-based evaluative approach in PM tasks could be more sensitive to the effects of MCI than traditional neuropsychological ones such as the Rivermead Behavioral Memory Test (RBMT; Wilson et al., 1985). Indeed, although RBMT was widely used in clinical settings, it does not include enough PM tasks to generate many types of performances and does not assess time-based PM performances (Mioni et al., 2014). VR, reaching a higher level of complexity, requires more cognitive resources to perform tasks, therefore could better represent the person in real life.

Based on this assumption, VEs are used to explore central theoretical questions about how the cognitive system successfully codifies and recovers intentional behavior (Gonneaud et al., 2014; Trawley et al., 2014). Gonneaud et al. (2014) assessed the impact of connections between the potential component (PC; remembering that something needs to be done) and the retention component (RC; the content of intention) of PM, in a Semi-Immersive (SI) urban environment where subjects could navigate using a virtual car. More specifically, the link between PC and RC affects the distinction between PM based on the appearance of an external cue (EB) and based on the automatic start of intention after a time interval (TB). Nine tasks were presented to the subjects: with a clear link between PC and RC (Link-EB; e.g., buying stamp book at a post office) or without (noLink-EB; e.g., buying eyeglasses at the fountain). Link-EB produced better performance than noLink-EB and TB, highlighting the importance of the association processes between PC and RC for effective PM. Similarly, Lecouvey et al. (2019) explored in VR the effects of mild AD on PM, showing that both the PM components are significantly compromised in AD patients, but RCs of intentions are altered before PCs. These data supported the hypothesis that early impairments of EM have a great impact on the execution of PM tasks in AD.

This VR approach is a more realistic tool that could help to better highlight planning processes, motivational aspects, time estimation, or eventual difficulties in dual-task processes in PM impairments in everyday life (Gonneaud et al., 2014; Lecouvey et al., 2019). Also, this approach could be useful to provide more efficient therapeutic interventions (Meijer et al., 2009) and a better measure of training effectiveness concerning less naturalistic performance. Indeed, VR technology has also been applied to cognitive training, such as in the paradigm of “Virtual Week” (Yip and Man, 2013; Rose et al., 2015). Mioni et al. (2015) outlined that VR improved PM performance in PD patients for the first time by using emotionally enriched tasks. Participants were asked to remember to carry out actions with positive value (e.g., “tell Roberta that Maria had a baby when you talk to Roberta”); with a negative value (e.g., “Pay a fine for speeding when you go shopping”) or with neutral value (e.g., “Buy your bus ticket after breakfast”). The tasks of PM with positive emotional value showed better results than tasks with negative or neutral value in both normal and PD patients, although the latter showed worse performance than the control group, independently of the emotional valence of the cue. Experimental data also provided improved outcomes in remembering to perform tasks with pleasant content compared to neutrals, since positive stimuli may attract more attention resources, hence facilitating the recovery and execution of PM actions. Furthermore, results seem to indicate that the use of a fully immersive task is feasible in the elderly: it arouses presence, it is addictive and causes limited symptoms of illness (Ouellet et al., 2018; Corriveau-Lecavalier et al., 2020).

However, it is important to note that due to technological limitations, state of art immersive environments do not correspond exactly to the real world. This contributes to the manifestation, in some users, of symptoms similar to those of the classic motion sickness called cybersickness, resulting from the conflict between the visual, vestibular, and proprioceptive sensory systems. Factors such as the previous familiarity with technology, age, or the presence of diseases can play an important role but, in particular, among older people, factors such as rotational speed and duration of exposure seem to increase cybersickness (Liu, 2014).

In the literature reviewed here, visual stimuli are presented mainly through SI systems, nevertheless, several studies applied fully immersive HMD systems (Nolin et al., 2013; Parsons and Barnett, 2017; Ouellet et al., 2018). A recent study conducted by Dong et al. (2016) compared desktop monitor activity and immersive VR activity with Oculus Rift to investigate whether traditional lab PM activity produced a similar result to PM activity in the VR environment. It was reported that while the performance of standard computer monitor tasks does not significantly correlate to the VR scores, a negative correlation between desktop reaction times and VR scores was observed, as well as a positive correlation between VR and desktop reaction times. VR seems to be more sensitive in accurately assessing PM in life. This is because the slide-based activity requires fewer cognitive resources than VR, where participants’ cognitive load has been increased and more correctly identifies worse scores when reaction times are slower. Notwithstanding, as pointed out by Plechatá et al. (2019), research should provide further clarification on the comparison between desktop platform performance and those in HMD, since different results in older people may be because participants have already had previous experiences with desktops but not with HMD platform that would lead to increased fatigue. Consequently, under certain circumstances, the level of immersion and a more complex context could be a problem for memory ability and participants may have limited cognitive resources for the memory task. Structural features such as movement or sensory feedback of the virtual environment can involve participants’ attention by draining cognitive energy because older adults unfamiliar with technology may find their management frustrating and this may distract from their virtual reality experience, making the task more demanding than for young adults.

Nonetheless, VR immersiveness is essential for exploiting the procedural involvement that allows us to predict the cognitive behavior of subjects as realistically as possible. In many of the described experimental environments, exploration took place while sitting, but people must navigate in the same way as they do in the real world (Tieri et al., 2018). The need arises from the fact that immersive VR navigation offers the user a much wider range of movement to approach or physically realistically interact with the virtual world, while the sitting position requires a set visual height, longer movements, and controller-based environment navigation. This freedom of movement is particularly problematic for VR-based PM studies that combine neuroimaging techniques (Dong et al., 2016) to ascertain changes in brain hemodynamic responses, and neuromodulation to improve PM performance in senior subjects (Debarnot et al., 2015).

Neurophysiological changes associated with VR neurorehabilitation can be measured using non-invasive and portable neuroimaging techniques, including fNIRS and/or EEG, equipped with a neuroergonomic and wireless approach, to measure cerebral blood flow in real-time during VR activity. Recently a study by Dong et al. (2017) investigated the function of the prefrontal cortex during a PM activity in an immersive VR environment *via* Oculus Rift and an OEG-16 multi-channel fNIRS system that allowed solving the problems often present for EEG such as the difficulty of movement for the application of electrodes. By using a virtual shopping experience, this study provided early confirmation of Brodmann area activation in a PM activity in VR but further studies are still needed to evaluate if and which other areas could potentially be involved during memory tasks in VE.

## Conclusions

The present review article provides a snapshot of virtual reality technology applications developed for assessment and improvement of episodic and/or PM, with the idea to suggest the integration of the most recent technological advancements to cognitive and aging neuroscience. It could be considered a flaw in our choice to also include studies employing young adults, though not strictly representative of the aging process. This inclusion, however, is based on a conceptual approach that recognizes that the study of aging must cover both older and younger populations trying to bridge what has been called a gap in geriatric research (Moffitt et al., 2017). The goal is to select additional evidence that currently cannot be obtained with adult subjects.

More importantly, it should be noted that the literature here reviewed included studies that used any form of VR technology, including non, semi, or totally immersive. It is important to note that the keywords of these reviews included the term “virtual reality” although this differs in terms of technical qualities and level of ecological validity. We believe that recognizing the centrality of the nomenclature in this field and the need for greater uniformity of language will create a more coherent and connected research field.

Also, the implementation of clinical VR research outside the laboratory still presents significant challenges that need to be addressed. The type of VR technology and the experimental designs implemented vary greatly between studies with many approaches using completely different hardware, software, or paradigms. However, the positive results provide reasons for more rigorously controlled research, necessary to progress from feasibility studies and pilot tests to standardized protocols that can be shared by the research community.

The literature here reviewed suggests that VR protocols offer an additional tool and excellent opportunity for innovative assessment and training options, particularly important in early identification of the subtle amnestic deficits that usually elude traditional methods. Some of the related advantages are the easy adaptability and the ability to replicate ecologically valid environments present in everyday life, allowing precise measurements of the cognitive processes involved. Furthermore, the possibility of providing a more stimulating context than in traditional laboratories can generate positive motivation in the elderly. However, the possible limitations associated with the perception of VR technology must be taken into account. The results of higher-immersive studies or greater interactivity are inconclusive in terms of the benefits of evaluating or training in the elderly population, particularly in pathological aging. Also, the introduction of the clinical population into VEs raises particular ethical and safety problems: some users experience health problems associated with the use of immersive HMD though these effects are mild and quickly fade. Susceptibility to cybersickness appears to be limited, but it could be related to short exposure times. Consequently, an in-depth research is needed to investigate how aging can affect motion sickness caused by immersive environments, to avoid the risk of reducing rather than increasing the ecological validity.

Finally, it is interesting to note that VR technology can be easily combined with other forms of technologies such as neuromodulation (tDCS) and neuroimaging (fNIRS/EEG) that can be considered valuable and indispensable tools to increase the benefits of VR. This perspective could provide a more targeted approach to neuro-training and will be the core of future research on the field.

## Author Contributions

AR and GG conducted the search and selection of bibliography. VR designed and directed the project. AR, GG, PS, and VR wrote the manuscript. All authors contributed to the article and approved the submitted version.

## Conflict of Interest

The authors declare that the research was conducted in the absence of any commercial or financial relationships that could be construed as a potential conflict of interest.
